# Thesis and dissertations examining tuberculosis in Brazil between 2013 and 2019: an overview

**DOI:** 10.1590/0037-8682-0198-2022

**Published:** 2022-08-12

**Authors:** Ana Júlia Reis, Juliana Lemos Dal Pizzol, Rúbia Gattelli, Andrea von Groll, Daniela Fernandes Ramos, Ivy Bastos Ramis, Afrânio Kritski, José Roberto Lapa e Silva, Pedro Eduardo Almeida da Silva

**Affiliations:** 1 Universidade Federal do Rio Grande, Faculdade de Medicina, Núcleo de Pesquisa em Microbiologia Médica, Rio Grande, RS, Brasil.; 2 Universidade Federal do Rio Grande, Biblioteca Setorial da Área Acadêmica da Saúde, Rio Grande, RS, Brasil.; 3 Universidade Federal do Rio de Janeiro, Faculdade de Medicina, Programa Acadêmico de Tuberculose, Rio de Janeiro, RJ, Brasil.

**Keywords:** Tuberculosis, Education, Professional education, Public health

## Abstract

**Background:**

Tuberculosis (TB) remains a serious public health problem, with approximately 10 million new cases reported annually. Knowledge about the quantitative evolution of theses and dissertations (T&Ds) examining human TB in Brazil can contribute to generating strategic planning for training professionals in this field and disease control. Therefore, this study highlights the role of T&Ds on TB in national scientific disclosures.

**Methods:**

An integrative review related to TB was performed, including T&Ds produced in Brazil and completed between 2013 and 2019.

**Results:**

A total of 559,457 T&Ds were produced, of which 1,342 were associated with TB, accounting for 0.24% of the total number of T&Ds in Brazil. This was evidenced by a predominance of themes such as attention/health care, epidemiology, and TB treatment, and 80.2% of the T&Ds on TB were related to the large areas of health and biological sciences. Only 19.7% of T&Ds were associated with groups of patients considered at risk for TB, and 50.9% were produced in southeastern Brazil. The 1,342 T&Ds on TB were developed in 416 postgraduate programs linked to 121 higher education institutions (HEIs). We highlight that 72.7% of T&Ds on TB were produced in federal HEIs, 27.4% in state HEIs, and 8.5% in private HEIs.

**Conclusions:**

Strategic themes, such as TB control, require public policies that aim to increase the number of doctors and masters with expertise in TB, with geographic uniformity, and in line with the priorities for disease control.

## INTRODUCTION

Tuberculosis (TB) remains a serious public health problem and is responsible for approximately 10 million new cases and 1.5 million deaths annually. Moreover, it is one of the main causes of death caused by a single infectious agent. Brazil is a priority country for this public health problem^1^, with approximately 90,000 new cases per year and a TB/human immunodeficiency virus (HIV) coinfection proportion of 11%^2^. 

At the World Health Assembly in 2014, the World Health Organization approved the End Tuberculosis Strategy. Their main objectives were to reduce 90% of TB cases and 95% of TB deaths by 2035. In addition, the strategy aims to eliminate or minimize the economic impact on families affected by TB^3^. In the following year, the United Nations launched the Sustainable Development Goals, which included a 90% reduction in deaths caused by TB by 2030^4^ ^,^ ^5^. 

Brazil's National Tuberculosis Control Program has used several strategies to control the disease, most of which are consistent with scientific evidence and guidelines recommended by the World Health Organization. This effort has resulted in improvements in epidemiological indicators, such as a reduction in the incidence and mortality of TB^6^ ^,^ ^7^. However, there are still many challenges, such as TB in prisons, TB/HIV coinfection, drug-resistant TB, other comorbidities (e.g., diabetes mellitus, mental health disorder, alcohol, illicit drugs, and tobacco use), a high proportion of treatment abandonment, low adherence to directly observed treatment, low contact evaluation, latent TB diagnosis and treatment, low coverage of rapid molecular diagnosis, and a low proportion of patients and family members who receive social protection^6^ ^,^ ^8^.

The success of actions that support global and national TB control and elimination strategies depends on qualified professionals generating, evaluating, and correctly using scientific knowledge. In Brazil, doctors and masters (D&M) are formed within the National Postgraduate System, whose Postgraduate Programs (PGPs) are accredited and periodically evaluated using the Coordination of Higher-level Personnel Improvement (CAPES) evaluation system^9^.

Knowledge about the quantitative evolution of theses and dissertations (T&Ds) produced in the area of human TB, as well as information about the spatiotemporal, thematic, and institutional distribution and its relationship with the TB burden in different populations and regions of Brazil, can contribute to generating strategic planning for the training of professionals in this theme. In addition, this study highlights the essential role of T&Ds in national scientific disclosures.

## METHODS

An integrative review was performed, including T&Ds related to TB, completed between January 1, 2013 and December 31, 2019, and made available in the CAPES database. This study was carried out based on the principles of scientometrics, which consist of *“Quantitative assessment and analysis of intercomparisons of activity, productivity, and scientific progress* ^10^.*”*

T&Ds identification and classification were performed independently by two researchers using the T&Ds catalog made available using the CAPES (Ministry of Education, Federal Government, Brazil) in Portuguese at https://dadosabertos.capes.gov.br/dataset.

The search for T&Ds was performed using the following Portuguese terms: *Mycobacterium tuberculosis, Mycobacteria*, antituberculostatics, isoniazid, and rifampicin. In addition, authorized descriptors in Portuguese, synonyms/alternative terms, related terms, and generic terms were used (DeCS/MeSH Health Sciences Descriptors - https://decs.bvsalud.org/) (Supplementary Material Table 1S). T&Ds that had any of these terms in the title, abstract, or keywords ^I^ were selected.

After the initial screening, the selected T&Ds were individually analyzed by two researchers, and those that mentioned any of the terms used in the search (Supplementary Material Table 1S) but whose work content was not associated with TB were excluded. The data were analyzed considering each thesis and dissertation equivalent to a doctorate and master's degree (professional and academic), respectively. In addition, the following variables were evaluated: T&Ds theme, the total number of T&Ds on TB produced in Brazil, period of time for D&M academic formation, CAPES assessment area, CAPES large knowledge areas, CAPES knowledge areas, subareas, risk groups for TB included in T&Ds, and T&Ds geographic and institutional distribution. 

According to the CAPES, the assessment areas are grouped into a large knowledge area, which in turn are grouped into knowledge areas and subareas (first level: large knowledge area: gathering of different knowledge areas, due to the affinity of their objects, cognitive methods, instrumental resources, and reflecting specific sociopolitical contexts; second level: knowledge area: set of interrelated knowledge, collectively constructed, gathered according to the nature of the investigation object, and for the purposes of teaching, research, and practical applications; and third level: subarea: segmentation of the knowledge area established according to the object of study and recognized and widely used methodological procedures)^11^.

T&Ds were classified into the following themes: attention/health care, biochemistry, diagnosis, drugs, epidemiology, genetics, immunology, resistance, and treatment. The classification was performed by searching for keyword^II^ in Portuguese associated with different themes (Supplementary Material List 1S). For T&Ds related to more than one theme, the main theme and its associated themes were independently defined by two researchers.

Data were tabulated in Microsoft Excel and analyzed using International Business Machine (IBM) Statistical Package for the Social Sciences (SPSS) software version 20.0 (International Business Machines Corporation **-** IBM - Armonk - New York - USA). The absolute and relative frequencies were determined.

## RESULTS

Between 2013 and 2019, considering all PGPs and knowledge areas, 559,457 T&Ds were produced in Brazil, of which 2,665 were initially selected as being associated with TB using the terms described in the Supplementary Material Table 1S. After an individual analysis, 1,342 T&Ds were selected for their association with TB, accounting for 0.24% of the total number of T&Ds produced in Brazil, of which 31.8% (427/1,342) were theses and 68.2% (915/1,342) were dissertations.

The total number of completed T&Ds in Brazil increased by 38.7% between 2013 and 2019, while the number of T&Ds on TB was proportionally reduced annually, beginning in 2014. When comparing 2013 and 2019, there was a 24.5% reduction in academic dissertations associated with TB, whereas the number of theses increased by 26%. Despite this, the number of theses concluded showed a 9% reduction between 2018 (the year with the greatest production) and 2019 (Table 1).

**TABLE 1:** Distribution of general theses and dissertations and those related to tuberculosis per year.

	Academic dissertations				Professional dissertations				Theses			
	General*		TB		General		TB		General		TB	
	n	%	n	%	N	%	n	%	n	%	N	%
2013	45,822	67.8	115	64.6	6,059	9.0	13	7.3	15,653	23.2	50	28.1
2014	46,370	65.6	127	65.1	7,012	9.9	15	7.7	17,286	24.5	53	27.2
2015	47,801	63.0	121	61.1	9,086	12.0	15	7.6	18,996	25.0	62	31.3
2016	49,055	61.1	95	48.0	10,618	13.2	41	20.7	20,605	25.7	62	31.3
2017	50,636	60.8	115	59.0	11,036	13.2	15	7.7	21,609	25.9	65	33.3
2018	52,068	59.1	103	52.3	13,125	14.9	23	11.7	22,927	26.0	71	36.0
2019	53,760	57.4	94	51.9	15,635	16.7	23	12.7	24,297	25.9	64	35.4

*Total production and percentage of each production type per year in Brazil. **Total productions and percentage of each type of production per year, associated with TB.

Between 2013 and 2019, there was a 77% increase in the number of professional dissertations. In 2016, professional master’s degrees represented 20.7% (41/198) of the T&Ds on TB produced in Brazil (Table 1). Of these, 48.8% (20/41) were carried out in Rio de Janeiro, with 85% (17/20) linked to the PGP of Family Health and Epidemiology in public health, coordinated by the Fundação Oswaldo Cruz (Fiocruz); 29.3% (12/41) were carried out in the state of Pernambuco, with 66.7% (8/12) linked to the PGP of public health of the Fiocruz.

When evaluating the necessary time to complete the postgraduate course, only approximately half of the T&Ds - doctorate 52.5% (224/427), academic master's degree 52.5% (404/770), and professional master's degree 48.3% (70/145) - were completed within 48 (doctorate) and 24 (master’s) months, the periods expected for presenting T&Ds in Brazil.

Regarding the large knowledge areas, among the 1,342 T&Ds produced during the study period, 67.9% and 12.3% were related to health sciences and biological sciences, respectively. The remaining T&Ds (19.8%) were multidisciplinary (8.4%), exact and earth sciences (6.8%), engineering (1.3%), applied social sciences (1.0%), human sciences (1.1%), agricultural sciences (1.0%), and linguistics, letters, and arts (0.3%).

When the knowledge areas were evaluated, 67.4% of the T&Ds associated with TB were concentrated in medicine (27.8%), public health (16.7%), and nursing (13.5%) (Table 2).

**TABLE 2:** Distribution of theses and dissertations on tuberculosis according to the CAPES knowledge areas and their corresponding assessment areas.

Knowledge area	N	%	Assessment areas
Medicine	162	12.1	Medicine I
Medicine	209	15.6	Medicine II
Medicine	2	0.15	Medicine III
Collective health	224	16.7	Collective health
Nursing	181	13.5	Nursing
Pharmacy	126	9.4	Pharmacy
Chemistry	83	6.2	Chemistry
General biology	60	4.5	Biological sciences I
Interdisciplinary	60	4.5	Interdisciplinary
Immunology	33	2.5	Biological sciences III
Biotechnology	26	1.9	Biotechnology
Microbiology	18	1.3	Biological sciences III
Biochemistry	17	1.3	Biological sciences II
Genetics	16	1.2	Biological sciences I
Education	13	1.0	Education
Parasitology	10	0.7	Biological sciences III
Environmental sciences	9	0.7	Environmental sciences
Electrical engineering	9	0.7	Engineering IV
Pharmacology	8	0.6	Biological sciences II
Others*	76	5.5	Others**
**Total**	**1,342**	**100.0**	

*administration (0.1% - 02/1,342); agronomy (0.4% - 06/1,342); botany (0.1% - 01/1,342); computer science (0.2% - 03/1,342); information science (0.1% - 02/1,342); food science and technology (0.1% - 01/1,342); political science (0.1% - 01/1,342); communication (0.1% - 01/1,342); ecology (0.1% - 01/1,342); economy (0.4% - 05/1,342); education (0.1% - 01/1,342); biomedical engineering (0.1% - 01/1,342); materials and metallurgical engineering (0.1% - 02/1,342); production engineering (0.1% - 02/1,342); mechanical engineering (0.1% - 01/1,342); nuclear engineering (0.1% - 01/1,342); chemical engineering (0.1% - 01/1,342); physics (0.3% - 04/1,342); geography (0.4% - 05/1,342); history (0.4% - 06/1,342); language (0.2% - 03/1,342); linguistics (0.1% - 01/1,342); mathematics (0.1% - 01/1,342); materials (0.4% - 05/1,342); veterinary medicine (0.4% - 05/1,342); morphology (0.1% - 01/1,342); nutrition (0.1% - 01/1,342); odontology (0.4% - 06/1,342); urban and regional planning (0.1% - 02/1,342); social service (0.1% - 01/1,342); sociology (0.1% - 02/1,342); zootechnics (0.1% - 01/1,342). **public and business administration, accounting science and tourism (0.1% - 02/1,342); astronomy/physics (0.3% - 04/1,342); biodiversity (0.1% - 02/1,342); computer science (0.2% - 03/1,342); food science (0.1% - 01/1,342); political science and international relations (0.1% - 01/1,342); agrarian sciences I (0,4% - 06/1342); biological sciences II (0.1% - 01/1,342); communication and information (0.2% - 03/1,342); economy (0.4% - 05/1,342); education (0.1% - 01/1,342); engineering II (0.3% - 04/1,342); engineering III (0.2% - 03/1,342); engineering IV (0.1% - 01/1,342,); geography (0.4% - 05/1,342); history (0.4% - 06/1,342); language/linguistics (0.1% - 01/1,342); linguistics and literature (0.2% - 03/1,342); mathematics/probability and statistics (0.1% - 01/1,342); materials (0.4% - 05/1,342); veterinary medicine (0.4% - 05/1,342); nutrition (0.1% - 01/1,342); odontology (0.4% - 06/1,342); urban and regional planning/demography (0.1% - 02/1,342); social service (0.1% - 01/1,342); sociology (0.1% - 02/1,342); zootechnics/fishing resources (0.1% - 01/1,342).

Almost all T&Ds within the knowledge area of medicine were related to the assessment areas of Medicine I and II. In the Medicine II assessment area, 209 T&Ds were concluded, with 38.8% on the infectious and parasitic diseases or tropical and infectious disease subareas. In the Medicine I assessment area, 162 T&Ds were concluded, with 53.7% distributed among the subareas of pulmonology, infectious diseases, and pneumological sciences. In addition to the subareas mentioned above, 26 other subareas were observed in the assessment area of Medicine I and 38 in Medicine II.

### Thematic classification

**FIGURE 1:**
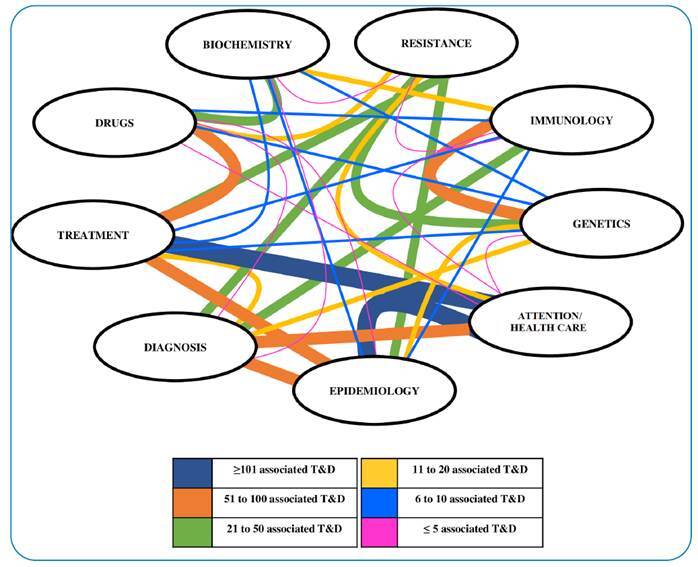
Intersections between the number of theses and dissertations with the main and associated themes. *Illustration made using *Microsoft PowerPoint* to visually demonstrate the relationship between themes.

T&Ds on TB were developed within a wide range of themes. Although 50.3% (675/1,342) of T&Ds could be classified as a single theme, 49.7% (667/1,342) were related to more than one theme, which was named “associated themes” in this study. With a predominance of themes, such as attention/health care, epidemiology, and treatment (Supplementary Material Table 2S), the association frequency between the main and associated themes was evaluated (Figure 1).

T&Ds on TB were also classified according to the type of study, with 50.1% related to basic research (drugs, genetics, immunology, resistance, and biochemistry), 33.9% to translational research (attention/healthcare), 30.5% to epidemiological research, 26.7% to TB treatment, and 19.4% to TB diagnosis.

### T&Ds associated with groups of patients at risk for tuberculosis development

Only 19.7% (264/1,342) of T&Ds cases were associated with groups of patients considered at risk for TB development (Table 3), with 5.7% (15/264) associated with more than one risk group.

**TABLE 3:** Number of theses and dissertations related to different groups of patients at risk for tuberculosis development and the prevalence of the disease among these groups in Brazil.

Risk groups	%	N	% in Brazil^&^
TB/HIV coinfection	8.2	110	11.0
Prisoners	2.9	39	8.9
Children and teenagers	2.5	34	8.4^#^
Indigenous individuals	1.6	21	1.0
Health professionals	1.5	20	1.0
Diabetes mellitus	1.3	18	7.2
Elderly people	1.2	16	14.1*
Homeless populations	0.8	11	3.0
Alcohol, drugs and/or tobacco users	0.8	11	3.9

**Source:** National Congress, Federal Government. BRASIL, 1990: According to the Child and Teenagers Statute, were considered children those aged up to 12 years and teenagers those aged up to 18 years. **Source:** National Congress, Federal Government. BRASIL, 2003: According to the Elderly Statute, all patients aged > 60 years were considered. ^&^Source: SINAN, 2021. Calculation based on the average per year of all TB cases in Brazil between 2013 and 2019 (89,104): ^#^ ^12^children and teenagers (< 1 year, 1-4, 5-9, 10-14, and 15-19 years); * ^13^and older adults (60-64, 65-69, 70-79, and ≥ 80 years).

### T&Ds geographical and institutional distribution

**FIGURE 2:**
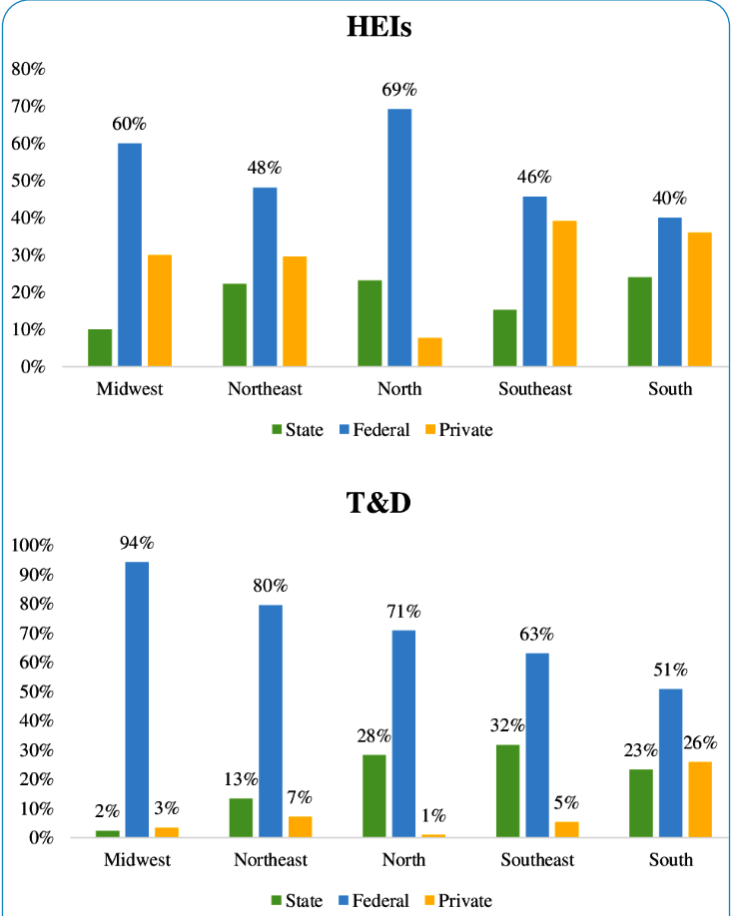
Distribution of higher education institutions, theses, and dissertations on tuberculosis in different regions of Brazil, according to the institution academic-administrative classification (federal, state, and private).

The 1,342 T&Ds on TB were produced in 416 PGPs and linked to 121 higher education institutions (HEIs) (Figure 2): 48.8% were federal, 19.8% were state, and 31.4% were private HEIs. Although approximately 1/3 (31.4%) of the HEIs were private, only 8.5% of the T&Ds on TB were produced in this type of institution, while 72.7% were in federal HEIs, and 27.4% were in state HEIs.

Overall, 50.9% of T&Ds on TB are produced in southeast Brazil. The state of Rio de Janeiro was responsible for 23% of T&Ds on TB produced in Brazil between 2013 and 2019, and 17.6% of T&Ds were produced in southern Brazil. Rio Grande do Sul produced 10% and 55.9% of T&Ds on TB in the country and southern Brazil, respectively. Finally, 16.8% and 8.2% of T&Ds were produced in the northeast and northern Brazil, respectively (Supplementary Material Table S3).

## DISCUSSION

Between 2013 and 2019, 0.24% of T&Ds produced in Brazil were associated with TB. According to a previous study, Brazil ranked sixth among countries that published the most on TB between 2007 and 2016, representing 3.8% of global publications associated with this theme^15^.

The total number of T&Ds in Brazil increased by 38.7% between 2013 and 2019, while the number of T&Ds on TB was proportionally reduced annually beginning in 2014, showing a reduction in the generation of D&M. This reduced formation of human capital in this area could put Brazilian TB control efforts at risk, harming the promotion, assistance, management, research, development, and innovation related to TB.

It is important to emphasize that the number of professional master's degree dissertations showed significant growth during the study period, which may be related to the fact that this is the newest modality of *stricto sensu* postgraduate study in recent years. For example, in 2016, the number of professional master's degrees represented 20.7% of the T&Ds on TB produced in Brazil, with a 77% increase when comparing 2013 and 2019.

In general, professional and academic master's degrees differ primarily in the formation of professionals who meet specific needs and have immediate applicability of the generated knowledge (professional) from those who will follow an academic career with a doctorate as their next goal (academic).

Regarding scholarships offered by the CAPES, there was an 11% increase in master's scholarships between 2011 and 2017 and a 23% increase in doctorate scholarships between 2010 and 2014^16^. Although there is a dissociation between the number of T&Ds on TB and the increase in scholarship offerings, the ratio between academic dissertations and theses decreased from 2.3 to 1.5 between 2013 and 2019, while the ratio between academic and professional dissertations decreased from 8.8 to 4.1 during the same period. These results indicate a policy to encourage the development of doctors and professional masters in relation to academic masters.

Although the CAPES recommended (before the coronavirus disease 2019 [COVID-19] pandemic) a maximum time for the training of masters and doctors of 24 and 48 months, respectively, only approximately half of the T&Ds on TB were completed within this time. Even though there is not necessarily a direct relationship between the deadline to complete the postgraduate course and the quality of T&Ds or the formation process, the time to complete a doctorate could be reduced through the implementation of strategies such as reducing the time for a master's degree to 1 year, as was recently proposed to the CAPES^17^.

As mentioned above, TB remains a serious public health problem in Brazil, with a high number of cases, a high proportion of TB/HIV coinfection, underreporting of cases (10-20% of undetected and/or untreated cases), high proportions of treatment abandonment, and a high incidence in vulnerable populations^1^ ^,^ ^6^ ^,^ ^18^. In this demanding scenario of needs and questions, T&Ds on TB were developed within a wide range of themes. There was a greater association between different themes, indicating an important multi-disciplinarity in the formation of D&M in the area of TB. This combination of themes boosts the formation of D&M with diverse skills and a broader view of problems.

Furthermore, according to previous studies^19^ ^,^ ^20^, T&Ds on TB were classified according to the type of study, with 50.1% related to basic research, 33.9% to translational research, 30.5% to epidemiological research, 26.7% to TB treatment, and 19.4% to TB diagnosis. A study evaluating publications on TB from BRICS countries (Brazil, Russia, India, China, and South Africa) indicated that 29.6% of publications were associated with epidemiological research, 33.8% with basic research, 13.1% with operational research, 10.1% with TB diagnosis, and 6.6% with TB treatment^15^.

When the groups of patients considered at risk for TB development were evaluated, we observed that only 19.7% of the T&Ds on TB were associated with these groups of patients, with 5.7% being associated with more than one risk group. Among the priority groups for TB control are people with positive serology for HIV (HIV+), prisoners, children/teenagers, indigenous individuals, health professionals, patients with diabetes mellitus, older adults, homeless populations, alcohol, drugs, and tobacco users^2^ ^,^ ^18^, only 8.2%, 2.9%, 2.5%, 1.6%, 1.5%, 1.3%, 0.8%, and 0.8% of T&Ds on TB were associated with these groups of patients, respectively. This dissonance between the main risk groups for TB development and the production of scientific knowledge, as well as the formation of professionals related to these essential issues, shows a precarious balance between academic needs and solutions, limiting the transfer of scientific benefits to the society. A significant number of TB cases in Brazil are among HIV+ patients, prisoners, children/teenagers, elderly people, and patients with diabetes mellitus^6^; however, only 16% of the T&Ds on TB were associated with these groups of patients.

Brazil is a continental country with profound inter- and intra-regional social, economic, educational, and public health asymmetries. This diversity of scenarios is also observed in relation to the T&Ds produced within the TB theme. Most T&Ds were concentrated in southeastern and southern Brazil and were produced in PGPs from public HEIs, particularly federal HEIs. The necessary impetus for the formation of D&M in regions such as northern and northeastern Brazil can be facilitated by public policies using connections established by the Brazilian Tuberculosis Research Network (REDE-TB)^21^.

In southeast Brazil, where 45.2% of TB cases occur, with a prevalence of 45.9 cases per 100,000 inhabitants, which is higher than that of the overall prevalence in Brazil (41.9 cases per 100,000 inhabitants), 50.9% of T&Ds on TB were produced. The state of Rio de Janeiro, with a TB prevalence of 79.5 cases per 100,000 inhabitants, was responsible for 23% of T&Ds on TB produced between 2013 and 2019. In southern Brazil, where 12.7% of TB cases occurred, with a TB prevalence of 37.4 cases per 100,000 inhabitants, 17.6% of T&Ds on TB were produced. Interestingly, 26% of T&Ds produced in southern Brazil were associated with private HEIs. The state of Rio Grande do Sul has approximately 50% of the HEIs and PGPs in southern Brazil and produces 10% and 55.9% of T&Ds on TB in the country and southern region, respectively^2^ ^,^ ^14^.

Northeast Brazil, which was home to 26.3% of TB cases in Brazil between 2013 and 2019, produced 16.8% of T&Ds on TB. Despite having 10.9% of TB cases with a prevalence of 51.6 cases per 100,000 inhabitants, Northern Brazil produced only 8.2% of the T&Ds on TB. Finally, we highlight the state of Amazonas, which has the highest TB prevalence in Brazil, with 81.2 cases per 100,000 inhabitants, and produces only 3.1% of the T&Ds on TB. In both regions, northeast and northern Brazil, unlike in southeast and southern Brazil, there is a dissonance between the TB burden and the number of D&M formed in the TB theme, which is likely related to the lowest number of PGPs in these regions^2^ ^,^ ^14^.

D&M can act in government institutions and civil society, both public and private, as a protagonist in the promotion, production, evaluation, and implementation of scientific knowledge. Scientific knowledge generation, which is necessary to overcome the challenges faced by society, depends on investments in infrastructure, the provision of money for research, and the formation of human capital. Furthermore, strategic themes, such as disease control, including TB, should be prioritized. Therefore, it is necessary to create public policies that can integrate PGPs, public health agencies, and entities representing civil society, among others, aiming for an expansion in the number of D&M with expertise in TB as well as a greater geographic uniformity in D&M formation, in line with the priorities for TB control.

Despite this, we highlight that a limitation of this study is related to the number and themes of T&Ds associated with TB may not reflect the quality of the research and product generation in Brazil, as this is evaluated through article impact factors and patent registration.

Finally, the development, evaluation, and implementation of new diagnostic platforms, more effective vaccines, new antimicrobials, evaluation of new therapies, and management strategies are essential and constitute the pillar of research and innovation in the End Tuberculosis Strategy. Despite this, the formation of D&M in knowledge areas with a technological profile that can meet the technological demands of the Brazilian Unified Health System has decreased, not exceeding 1/5 of the titled D&M. This scenario puts efforts for TB control at risk and jeopardizes technological sovereignty promotion, foreign exchange savings, and the universalization of academic knowledge benefits^22^.

## SUPPLEMENTARY MATERIAL

**LIST S1: KEYWORDS USED TO CLASSIFY THESES AND DISSERTATIONS IN THEMES.**

**ATTENTION/HEALTH CARE:** healthcare access; assistance; attention to the person with tuberculosis; attitudes and practices; basic health care; basic health indicators; basic health unit/units; brief intervention; capacity building; collective health; committee methods; communicators; community councils; community health agent; comprehensive health care; contacts; control measures; control program; cost/costs; cost-effectiveness; disease control; educational material/materials; educational technology; interventions effectiveness; family health; fight against tuberculosis; healthcare promotion; healthcare; health communication; health conditions analysis; health education; health evaluation; health policies; health service/services; health surveillance; health teaching; health training; health unic system; health unit/units; home contact; home visit; income; inequalities/inequality; infection control; information source; information system/systems; integrated management; knowledge about tuberculosis; knowledge evaluation; living condition/conditions; management; meanings and experiences; medical education; nurses' actions/appointment; nursing practice; health professionals' performance; permanent education; pharmaceutical attention; post-discharge management; prevention and control; primary health care; public health; public health policies; service qualification; life quality; information quality; reference center/laboratory/unit; referral hospital/service; referral outpatient clinic; respiratory symptomatic; route; sanitary service; social condition/conditions; social determinants; social development; social indicators; social inequity; social mobilization; social protection; social representations; social security; social support; stigma; trajectories/trajectory; tuberculosis control; tuberculosis indicators; undernotification; vulnerability.

**BIOCHEMISTRY:** acetyltransferase/acetyltransferases; ATP: biochemicals; biochemistry; cathepsin; enzymatic; enzyme/enzymes; glycation; leptina; lipid mediators; metalloproteinase/metalloproteinases; oxidative stress; phospholipase; protease; proteolysis; reductase/reductases; shikimate; transferase; transpeptidase/transpeptidase.

**DIAGNOSIS:** accuracy; bacilloscopy/BAAR; biomarker/biomarkers; clinical decision; clinical laboratory; detection; diagnosis; diagnoses/diagnosis; GeneXPERT/XPERT; signs and symptoms identification; IGRA; immunodiagnostic; molecular rapid test; mpt64; multiplex/multiplex PCR; mycobacteria identification; point-of-care; quantiferon/quantiferon-TB; radiography; screening exam; tomographic features; tuberculin proof; tuberculin test/skin test; X-ray.

**EPIDEMIOLOGY:** age/age and sex effects; associated determinants/factors; association measures; case analysis; case-control; clinical and laboratory profile; clinical-epidemiological; determinant factors; epidemiological; epidemiology; genotyping; geoepidemiological; geographic aspects; health geography; hospitalization time; incidence; lethality; MIRU/MIRU-VNTR; molecular characterization; morbidity; mortality; notification; notified; pharmacoepidemiological; prevalence; probabilistic correlation; related factors; risk factor/factors; score; spatial analysis/distribution; spatio-temporal; sociodemographic; socioeconomic; spoligotyping; survival; systematic review; temporal analysis.

**GENETICS:** alleles; beijing; chemogenomics; epigenetic/epigenetics; gene/genes; gene expression; gene transcription; genetic/genetics; genome; genotype/genotypes; hemeproteins; lineage/lineages; metabolomics; microRNA; mutation/mutations; polymorphism/polymorphisms; protein/proteins; protein subunit; proteomics; transcriptional regulation; transcriptional repressor; sequencing; sub-lineage.

**IMMUNOLOGY:** antibodies; antigen/antigens; apolipoprotein; BCG; CD cells; cytokine/cytokines; dendritic cells; epitopes; granuloma; HLA; humoral response; IFN; immune; immunodiagnostic; immunological; immunomodulation; immunomodulator/immunomodulators; immunomodulatory; immunopathogenesis; immunopathology; inflammatory markers; inflammasome/inflammasomes; interleukin/IL; interferon; lymphocyte/lymphocytes; lysosome; macrophage; monocytes; neutrophils; T-cells; toll-like; tumor necrosis factor/TNF; vaccine/vaccines.

**DRUGS:** activity evaluation; anti-*Mycobacterium tuberculosis* activity/activities; antimicrobial action/activity/potential; antimycobacterial activity/potential; antituberculosis activity; biocomposites; biological activity/activities; biological evaluation; biological prospecting; bioprospecting; biosynthesis; chemical and biological study; computational modeling; computational simulation; cytotoxicity; cytotoxicity; cytotoxicological evaluation; drug development; drug planning; extract/extracts; formulation/formulation development; inhibitor development; medicine innovation; microparticles; nanocarriers; nanocomposite/nanocomposites; nanofibers; nanomaterials; nanoparticle/nanoparticles; nanoreservoir; new derivatives; new drugs/molecules; obtaining and characterization; pharmaceutical excipient; pharmaceutical innovations; pharmacokinetic studies; pharmacophore; pharmacophoric; phytochemical study; physical stability; physicochemical characterization; potential action; potential activity; randomized clinical trial; rational drug design; molecule reuse; secondary metabolites; solubility; drug structural characterization; structure activity; synthesis; synthetic and antimicrobial potential; tuberculostatic activity.

**RESISTANCE:** antibiofilm; biofilm/biofilm; drug resistant; efflux; MDR-TB: minimal inhibitory concentration; modulatory effect: monoresistant; multidrug resistant/resistance; multi-resistant; resistance to drugs; sensitivity test/tests.

**TREATMENT:** abandonment; acetylation; adverse reactions; anti-tuberculosis drugs; anti-tuberculosis therapy; anti-tuberculosis regimens; bacteriological conversion; blood concentrations; case outcomes; chemoprevention; chemoprophylaxis; clinical/clinical and therapeutic evolution; combined therapy; cure; directly observed therapy; drug exposure; drug interaction/interactions; drug therapy; fixed combined dose; hepatotoxicity; isoniazid; quadruple scheme; plasmatic concentrations; post-treatment; medicines rational use; retreatment; rifampicin; serum concentrations; therapeutic adherence; therapeutic effect; therapeutic intervention; therapeutic itinerary/itineraries; therapeutic outcomes; therapeutic scheme; treaties; treatment; tuberculosis therapy.

## Appendix

**Supplementary Material Table 1S:** Health sciences descriptors.

Term	Descriptor in Portuguese	Synonyms	Related terms	Generic terms
Tuberculosis	Tuberculosis	*Mycobacterium tuberculosis* infection	Antituberculous Interferon-gamma release tests	*Mycobacterium* infections
		Sanitary pulmonology TB	Tuberculin test	Communicable diseases
*Mycobacterium tuberculosis*	*Mycobacterium tuberculosis*	*Mycobacterium tuberculosis*	Antituberculous	*Mycobacterium*
*M. tuberculosis*		H37Rv		
*Mycobacteria*	*Mycobacterium*	Mycobacteria		*Mycobacteriaceae*
Antituberculostatics	Antitubercular Agents	Agent, Anti-Tuberculosis	Tuberculostatics	Antibacterials
			Multiple drug resistant tuberculosis	
		Agent, Antitubercular		
		Agent, Tuberculostatic		
		Agents, Antitubercular		
		Agents, Tuberculostatic		
		Antitubercular Drug		
		Anti Tuberculosis Drugs		
		Anti Tuberculosis Drug		
		Antitubercular Drug		
		Antitubercular Drugs		
		Drug, Anti-Tuberculosis		
		Drug, Antitubercular		
		Drugs, Anti-Tuberculosis		
		Drugs, Antitubercular		
		Tuberculostatic Agent		
		Tuberculostatic Agents		
Isoniazid	Isoniazid	Isonicotinic acid hydrazide		Hydrazines
				Isonicotinic acids
Rifampicin	Rifampicin	Rifampicin		Rifamycins

## Appendix

**Supplementary Material Table 2S:** Main and associated themes

	Main theme*	Associated theme*	Total	
	N	N	N	%
Attention/health care	253	205	458	33.9
Epidemiology	271	138	409	30.5
Treatment	165	193	358	26.7
Diagnosis	160	101	261	19.4
Drugs	198	15	213	15.8
Genetics	57	90	147	10.9
Immunology	106	16	122	91
Resistance	74	41	115	8.6
Biochemistry	36	40	76	5.7
Others**	122			

*****Based on total T&D. ******T&D not classified in any of the themes.

## Appendix

**Supplementary Material Table 3S:** Theses and dissertations geographical and institutional characterization

Federated Unit	Theses and dissertations	%	Prevalence by 100,000 inhabitants	Higher education institutions	Postgraduate Programs
Southeast					
Rio de Janeiro	303	22.6	79.5	11	67
São Paulo	243	18.1	45.0	20	81
Minas Gerais	103	7.7	19.4	14	40
Espírito Santo	34	2.5	33.5	1	5
Total	683	50.9	45.1	46	193
Northeast					
Pernambuco	58	4.3	59.2	6	17
Bahia	42	3.1	36.9	4	14
Paraíba	37	2.8	33.6	4	8
Ceará	39	2.9	45.4	4	14
Rio Grande do Norte	21	1.6	36.9	1	7
Maranhão	18	1.3	33.6	3	8
Piauí	4	0.3	25.3	2	2
Sergipe	4	0.3	36.1	1	3
Alagoas	3	0.2	37.2	2	3
Total	226	16.8	40.7	27	76
Southern					
Rio Grande do Sul	132	9.8	57.5	12	37
Paraná	70	5.2	22.1	9	28
Santa Catarina	34	2.6	30.5	4	13
Total	236	17.6	37.4	25	78
Northern					
Pará	56	4.2	50.4	5	18
Amazonas	42	3.1	81.2	3	15
Rondônia	7	0.5	39.6	1	3
Roraima	2	0.1	31.8	1	1
Acre	1	0.1	51.1	1	1
Amapá	1	0.1	30.1	1	1
Tocantins	1	0.1	12.6	1	1
Total	110	8.2	51.6	13	40
Midwest					
Distrito Federal	30	2.3	15.2	3	10
Goiás	27	2.0	15.3	3	10
Mato Grosso do Sul	27	2.0	41.5	3	7
Mato Grosso	3	0.2	43.9	1	2
Total	87	6.5	25.9	10	29
Brazil	1,342	100.0	41.9	121	416
